# 2880. Propensity score-weighted analysis of the impact of outpatient parenteral antimicrobial therapy (OPAT) plan reconciliation on unscheduled care

**DOI:** 10.1093/ofid/ofad500.157

**Published:** 2023-11-27

**Authors:** William D Sieling, Jennifer Ross, Michael Evans, Kaylyn Billmeyer, Elizabeth B Hirsch, Susan E Kline, Alison Galdys

**Affiliations:** University of Minnesota Medical School, Minneapolis, MN; M Health Fairview - University of Minnesota Medical Center, Minneapolis, MN; University of Minnesota, Minneapolis, Minnesota; University of Minnesota College of Pharmacy, Minneapolis, Minnesota; University of Minnesota College of Pharmacy, Minneapolis, Minnesota; University of Minnesota Medical School, Minneapolis, MN; University of Minnesota, Minneapolis, Minnesota

## Abstract

**Background:**

Outpatient parenteral antimicrobial therapy (OPAT) is a mechanism for delivery of antimicrobial therapy over a prolonged period in an environment outside of inpatient care. Benefits of OPAT include avoidance of hospital stays, prevention of hospital-associated conditions, and significant cost savings. We analyzed the frequency of all-cause 90-day emergency department (ED) visits, readmissions, and mortality of patients pre- and post-implementation of OPAT plan reconciliation in June of 2020.

**Methods:**

Unique, adult OPAT recipients discharged to home or post-acute care facilities from an academic hospital between 6/2017 and 6/2022 were included in our cohort. On 6/14/2020, a program was launched that entailed OPAT plan review and reconciliation by infectious diseases (ID) pharmacists prior to OPAT recipients discharging from acute care; all participants discharged on or after this date were included in the post-intervention cohort. (Figure 1) Data on patient characteristics, admission events, and outcomes were collected from the electronic medical record. We performed a propensity score weighted analysis with pre-defined variables to determine which demonstrate an association with our outcomes. We accounted for missing data by using multiple imputation.

**Figure:**
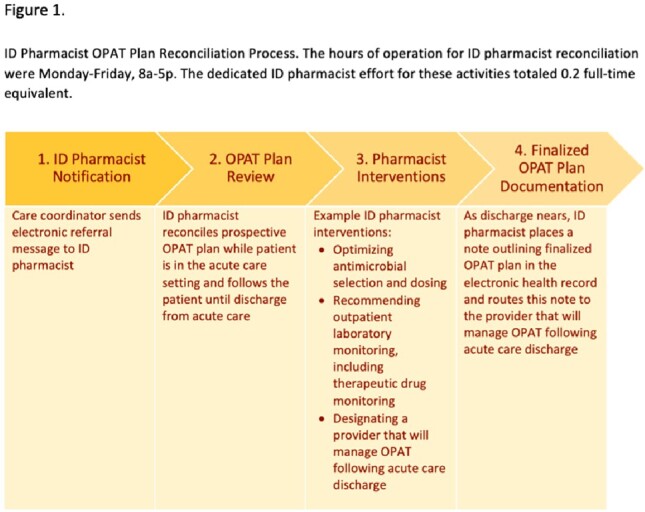

**Results:**

2408 OPAT patients meeting inclusion criteria were identified: 1650 pre-implementation and 758 post-implementation. (Table 1) Variables for which the standard mean difference between groups was ≥ 0.1 include race, chronic kidney disease, length of stay, ID consultation, and payor. (Figure 2) In our propensity-weighted analysis, there was a statistically significant difference between the proportion of patients in the pre- and post-implementation groups that presented to the ED (pre- 22.3%; post- 17.9%; p=0.032) or were readmitted (pre- 39.1%; post- 33.1%; p=0.008) within 90 days after discharge from index admission. (Table 2) There was no significant difference in the 90-day all-cause mortality between the pre- and post-implementation cohorts.

**Figure:**
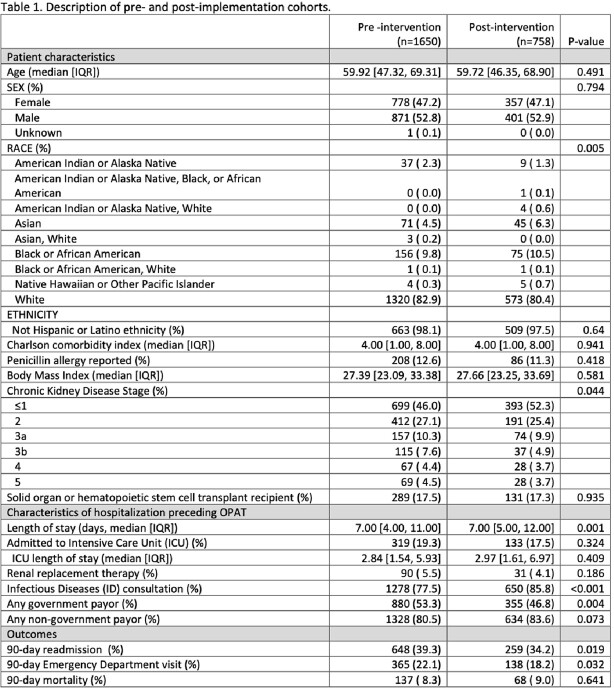

**Figure:**
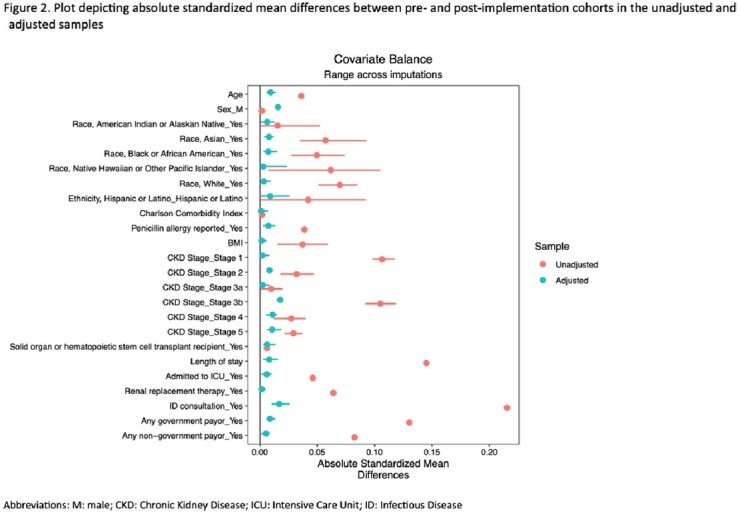

**Figure:**
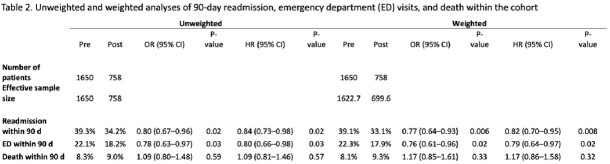

**Conclusion:**

Following institution of OPAT plan reconciliation by ID pharmacists prior to discharge from acute care, OPAT recipients were significantly less likely to experience 90-day ED visits or 90-day readmissions.

**Disclosures:**

**Elizabeth B. Hirsch, PharmD**, Melinta Therapuetics: Honoraria|Merck and Company, Inc.: Grant/Research Support

